# Mannich base-connected syntheses mediated by *ortho*-quinone methides

**DOI:** 10.3762/bjoc.14.43

**Published:** 2018-03-06

**Authors:** Petra Barta, Ferenc Fülöp, István Szatmári

**Affiliations:** 1 Institute of Pharmaceutical Chemistry and Research Group for Stereochemistry, Hungarian Academy of Sciences, University of Szeged, Eötvös u. 6, H-6720 Szeged, Hungary

**Keywords:** aminophenols, [4 + 2] cycloaddition, dienophile, Mannich reaction, *ortho-*quinone methide

## Abstract

This article provides an overview about specifically modified Mannich reactions where the process involves an *ortho*-quinone methide (*o*-QM) intermediate. The reactions are classified on the basis of the *o*-QM source followed by the reactant, e.g., the dienophile partner in cycloaddition reactions (C=C or C=N dienophiles) or by the formation of multicomponent Mannich adducts. Due to the important pharmacological activities of these reactive *o*-QM intermediates, special attention is paid to the biological activity of these compounds.

## Review

### Introduction

The Mannich reaction is an important, one-pot, multicomponent, C–C bond forming reaction that is widely used in the syntheses of many biologically active and natural compounds [1–5]. Originally, the Mannich product is formed through a three-component reaction containing a C–H acid, formaldehyde and a secondary amine. Recently, one of its special variations called modified Mannich reaction, has gained ground, in which the C–H acid is replaced by electron-rich aromatic compounds such as 1- and 2-naphthols as active hydrogen sources [6]. At the beginning of the 20th century, Mario Betti reported the synthesis of 1-aminobenzyl-2-naphthol starting from ammonia, benzaldehyde and 2-naphthol. This protocol is known as Betti reaction and the compound formed as Betti base [7–9]. Several examples have been published to extend the reaction and to synthesize varied substituted aminonaphthol derivatives [10]. Their relatively easy accessibility and promising biological properties have led to the resurgence of their chemistry coming again into the focus of pharmacological research.

The formation of aminonaphthols can be explained by two mechanisms. According to one possibility, first the reaction of the amine and the aldehyde yields a Schiff base and then the latter reacts with 2-naphthol in the second nucleophilic addition step. The other theory assumes the formation of an *ortho*-quinone methide (*o*-QM) intermediate by the reaction of 2-naphthol and benzaldehyde. Re-aromatization, the driving force of the transformation, takes place in the second step by the nucleophilic addition of the amine component.

The class of *o*-QMs has recently been investigated from many aspects. They are known as short-lived species playing an important role as key intermediates in numerous synthetic pathways. Reviews have recently been published about *o*-QM generation, applicability in organic syntheses and biological properties [11–17]. However, in this review we would like to focus on their role in syntheses connected to Mannich base chemistry as well as their wider applicability and properties.

### Formation of Mannich bases via *o-*QM intermediates

#### Synthesis of amidoalkylnaphthols

The preparation of amidoalkylnaphthols has recently been discussed from many points of view [18]. This indicates the importance of this reaction because 1-amidoalkyl-2-naphthols can be easily converted to important biologically active 1-aminoalkyl-2-naphthol derivatives by a simple amide hydrolysis.

**Scheme 1:**
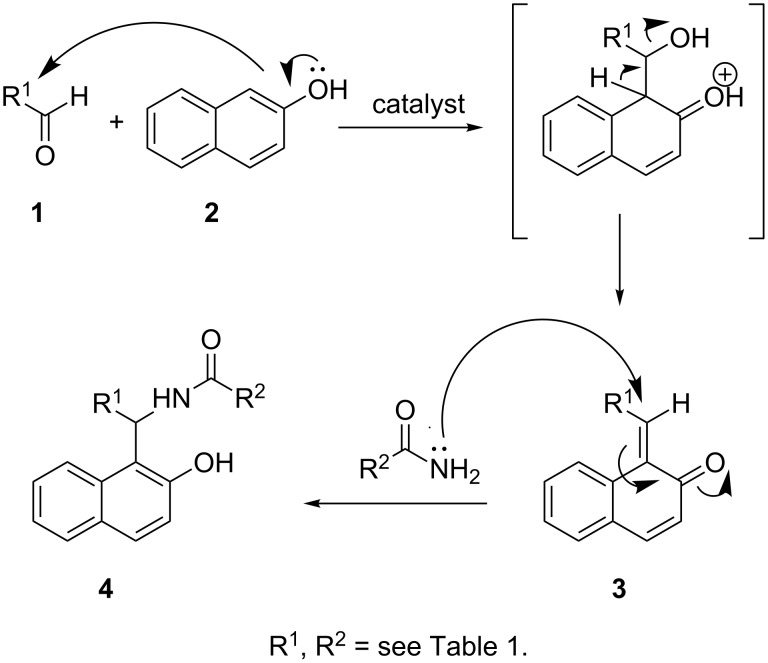
Formation of amidoalkylnaphthols **4** via *o*-QM intermediate **3**.

The mechanism of the Mannich reaction is depicted in Scheme 1. First, the reaction between the aldehyde and 2-naphthol, induced by the catalyst, leads to the generation of *o*-QM intermediate **3** that reacts further with the amide component to form the desired 1-amidoalkyl-2-naphthol derivatives. This second step can also be considered as a nucleophilic addition of the amide to the *o*-QM component.

Various catalysts and conditions were used to optimize reaction conditions considering economical and environmental aspects. These include microwave-assisted reactions, solvent-free conditions and the reusability of the catalyst (Table 1). Procedures are carried out as one-pot multicomponent transformations without the isolation of the intermediates formed. Therefore, with the application of nontoxic, readily available and inexpensive reagents, both time and energy are saved.

**Table 1:** Comparison of various conditions applied in the synthesis of amidoalkylnaphthols **4**.

entry	R^1^	R^2^	catalyst	conditions	yields (%)	ref.

1	Ph, 2,4-Cl_2_Ph, 3-BrPh, 4-NO_2_Ph, 4-(CHO)Ph, 5-Br-2-OHPh, 2-pyridyl, 4-MePh, Et, 3-OEt-4-OHPh, 4-OHPh, 4-OMePh, 3-BrPh	Me, Ph, NH_2_	ASA NPs	80 °C, 8–30 min	67–96	[19]
2	Ph, 2-ClPh, 4-ClPh, 4-BrPh, 4-MePh, 4-OMePh, 3-NO_2_Ph, 4-NO_2_Ph, 4-CNPh, 3-OMePh, 1-Nph	Me, NH_2_	nano-sulfated zirconia	120 °C, 32–85 min	81–94	[20]
3	Ph, 4-MePh, 4-ClPh, 4-OMePh, 4-NO_2_Ph, 3-NO_2_Ph, 2-ClPh, 2-NO_2_Ph, 2-MePh, 3-OMePh	Me	PbS nanoparticles	120 °C, 4–9 min	85–95	[21]
4	Ph, 4-MePh, 4-NMe_2_Ph, 4-OMePh, 3-NO_2_Ph, 4-FPh, 2,4-Cl_2_Ph, 2,5-(OMe)_2_Ph, 3,4-(OMe)_2_Ph, 2,3-(OMe)_2_Ph, 2-ClPh, 2-NO_2_Ph, 3-OH-4-OMePh, 2-FPh, C_10_H_7_, Et, Pr	Me, Ph, NH_2_	MNPs–SO_3_H	100 °C, 7–35 min	77–97	[22]
5	Ph, 4-NO_2_Ph, 4-ClPh, 2,5-(OMe)_2_Ph, 2-furyl, 2-thiophene, 1-Nph, 2-Nph, C(Me)=CH-Ph, CH=CH-Ph, Ph-Ph	Me, Ph, NH_2_	{[HMIM]C(CN)_3_}	rt, 5–30 min	90–96	[23]
6			nano SnO_2_	rt, 17–35 min	81–88	[23]
7	Ph, 2-ClPh, 4-ClPh, 2,6-Cl_2_Ph, 4-BrPh, 3-NO_2_Ph, 4-NO_2_Ph, 3-MeOPh, 4-MePh, 2,5-(OMe)_2_Ph, 4-CNPh, 4-AcPh	Me, Ph, NH_2_	[TEBSA][HSO_4_]	120 °C, 10 min	73–91	[24]
8	Ph, 4-ClPh, 4-OMePh, 4-MePh, 2-furyl, 2-thiophene, 3-formylchromone	Me, Ph, NH_2_, OEt	EAN	rt, 60 min	85–96	[25]
9	Ph, 3-NO_2_Ph, 4-OHPh, 4-OMePh, 2-ClPh, 4-ClPh, 4-NO_2_Ph, 4-NMe_2_Ph, 3,4,5-(OMe)_3_Ph	Me, Ph, NH_2_	SILC	100 °C, 7–10 min	80–95	[26]
10	Ph, 2-ClPh, 4-ClPh, 2-OMePh, 4-OMePh, Et, Pr	Me, Ph, NH_2_	CFBILs	70 °C, 25–60 min	84–94	[27]
11	Ph, 4-MePh, 4-OMePh, 4-NMe_2_Ph, 3-NO_2_Ph, 2,4-Cl_2_Ph, 2-ClPh, 2-NO_2_Ph, 2,3-(OMe)_2_Ph, C_10_H_7_, Pr	Me, Ph, NH_2_	MNP-IL-OAc	100 °C, 60–210 min	82–97	[28]
12			MNP-IL-OAc	sonication, 10–30 min	90–98	[28]
13	Ph, 4-ClPh, 3-NO_2_Ph, 4-BrPh, 4-MePh, 3-NO_2_Ph, 2-ClPh, 2,4-Cl_2_Ph	Me, Ph, NH_2_, NHMe, vinyl	*p*-TSA	DCE, rt, 9–30 h	83–96	[29]
14			*p*-TSA	125 °C, 4–10 h	80–95	[29]
15	Ph, 2,4-Cl_2_Ph, 3-OMePh, 3-NO_2_Ph, 3,4-(OMe)_2_Ph, 4,5-(OMe)_2_-2-NO_2_Ph, 4-BrPh, 3,4,5-(OMe)_3_Ph, 2-pyridyl, 3-indolyl, 2-furyl	Me, Ph, NH_2_	montmorillonite K10	125 °C, 30–120 min	65–96	[30]
16	Ph, 3-NO_2_Ph, 4-ClPh, 4-BrPh, 4-MePh, 4-FPh, 2-BrPh, 2-MePh, 2-ClPh, 3-BrPh	Me, Ph, NH_2_, NHMe, vinyl	Indion-130	110 °C, 6–30 min	81–94	[31]
17	Ph, 4-MePh, 4-ClPh, 3-NO_2_Ph, 2-BrPh, 2,4-Cl_2_Ph, 1-Nph, Et	Ph, NH_2_, NHMe	I_2_	DCE, 125 °C, 10–26 h	35–93	[32]
18			I_2_	125 °C, 4–9 h	20–90	[32]
19	Ph, 4-BrPh, 2-ClPh, 4-ClPh, 2,4-Cl_2_Ph, 3-OMePh, 3-NO_2_Ph, 4-MePh	Me, Ph, NH_2_	K_5_CoW_12_O_40_·3H_2_O	125 °C, 2–6 h	74–88	[33]
20			K_5_CoW_12_O_40_·3H_2_O	DCE, rt, 10–24 h	83–92	[33]
21	Ph, 4-BrPh, 2-ClPh, 4-ClPh, 4-FPh, 4-CNPh, 3-OMePh, 3-NO_2_Ph, 4-MePh	Me, Ph, NH_2_	HClO_4_-SiO_2_	DCE, 125 °C, 6–8 h	85–92	[34]
22			HClO_4_-SiO_2_	125 °C, 8–14 min	90–96	[34]
23	Ph, 4-BrPh, 4-ClPh, 4-FPh, 4-MePh 4-NMe_2_Ph, 4-NO_2_Ph, 4-OMePh, 3-NO_2_Ph, 3-FPh, 3-OMePh, 2,4-Cl_2_Ph, 2,5-(OMe)_2_Ph, 3,4-(OMe)_2_Ph, 2-ClPh, 2-NO_2_Ph, 2-MePh	Me, Ph	HClO_4_-SiO_2_	ACN, 85 °C, 20 h	60–88	[35]
24			HClO_4_-SiO_2_	110 °C, 30–80 min	76–91	[35]
25			HClO_4_-SiO_2_	MW 450 W, 12–20 min	75–94	[35]
26	Ph, 4-ClPh, 4-FPh, 3-CF_3_Ph, 3-NO_2_Ph, 4-MePh, 4-EtPh, 4-OHPh, 4-MeOPh, 3-OMe-4-OHPh, Et, iPr	Me, Ph, NH_2_, vinyl	HClO_4_-SiO_2_	125 °C, 5–9 h	68–93	[36]
27	Et, iPr, CH=CHPh, C_5_H_10_, cyclohexyl, 2-pyridyl, Ph, 4-ClPh, 3-OMePh, 4-pyridyl	Me, Bn	SSA	rt, 1.5–2.5 h	79–85	[37]
28	Ph, 4-ClPh, 2-MePh, 2-ClPh, 3-NO_2_Ph, 4-FPh, 4-MePh, 4-BrPh, 2-OMePh, 3-OMePh	Me	ClSO_3_H	ACN, 85 °C, 3 h	90–98	[38]
29	Ph, 4-MePh, 4-NO_2_Ph, 4-NMe_2_Ph, 4-ClPh, 4-BrPh, 4-OMePh, 3-NO_2_Ph3-FPh, 4-FPh, 2,4-Cl_2_Ph, 2,5-(OMe)_2_Ph, 2-ClPh, 3-OMePh, 2-NO_2_Ph, 2-MePh, 3,4-(OMe)_2_Ph	Me	NaHSO_4_·H_2_O	ACN, 85 °C, 20 h	65–88	[39]
30			NaHSO_4_·H_2_O	120 °C, 7–40 min	77–94	[39]
31			NaHSO_4_·H_2_O	MW 800 W, 3–14 min	73–91	[39]
32	Ph, 4-NMe_2_Ph, 4-OMePh, 4-ClPh, 4-BrPh, 3-NO_2_Ph, 4-FPh, 2,4-Cl_2_Ph, 2-ClPh, 3-OMePh, 2-NO_2_Ph, 3-OMePh, 2-NO_2_Ph, 2-MePh, 3,4-(OMe)_2_Ph, 4-MePh, 4-NO_2_Ph, 3-FPh, 2,5-(OMe)_2_Ph	Me, Ph	Fe(HSO_4_)_3_	ACN, 85 °C, 20 h	51–74	[40]
33			Fe(HSO_4_)_3_	85 °C, 25–80 min	74–97	[40]
34			Fe(HSO_4_)_3_	MW 450 W, 5–14 min	84–96	[40]
35	Ph, 4-OMePh, 4-MePh, 4-ClPh, 3-NO_2_Ph, 2-furyl	Me, Ph, NH_2_, vinyl, 2-thiophenyl	Sr(OTf)_2_	CHCl_3_, 60 °C, 8–15 h	80–96	[41]
36	Ph, 4-OMePh, 4-MePh, 4-ClPh, 4-NO_2_Ph, 3-NO_2_Ph, 2,4-Cl_2_Ph, 2-ClPh, 2-MePh	Me, Ph	CuPW	Bu_4_NBr, 100 °C, 90 min	74–95	[42]
37			CuPMo	Bu_4_NBr, 100 °C, 90 min	70–93	[42]
38	Ph, 4-ClPh, 4-BrPh, 4-FPh, 4-CNPh, 3-NO_2_Ph, 3-OMePh, 2-ClPh	Me, Ph, NH_2_	wet-TCT	100 °C, 8–14 min	90–96	[43]
39	Ph, 3-NO_2_Ph, 4-ClPh, 2-MePh, 2-ClPh, 4-FPh, 3-NO_2_Ph, 4-BrPh, 3-BrPh, 2-BrPh, 2-furyl	Me	sulfamic acid	sonication, 28–30 °C, 10–60 min	55–92	[44]
40			sulfamic acid	sonication, DCE, 28–30 °C, 25–120 min	78–94	[44]
41	Ph, 4-ClPh, 4-OMePh, 3-NO_2_Ph, 2-furyl, 2-ClPh, Et	Me, Ph, NH_2_, vinyl	I_2_	DCE, rt, 8–24 h	30–93	[45]
42	Ph, 4-NO_2_Ph, 3-NO_2_Ph, 2-NO_2_Ph, 4-ClPh, 2-ClPh, 4-OMePh, 2-OMePh, 4-MePh, 2,4-Cl_2_Ph, 4-NMe_2_Ph	Me, Ph	P_2_O_5_	60 °C, 5–15 min	80–97	[46]
43	Ph, 4-NO_2_Ph, 3-NO_2_Ph, 2-NO_2_Ph, 4-CN-Ph, 4-FPh, 3-FPh, 4-BrPh, 2-ClPh, 2,4-Cl_2_Ph, 4-ClPh, 2-MePh, 4-MePh, 3-OMePh, 4-OMePh, 3,4-(OMe)_2_Ph, CH=CH-Ph	Me, Ph	P_2_O_5_·SiO_2_	100 °C, 3–40 min	54–94	[47]
44	Ph, 4-OHPh, 4-ClPh, 2-ClPh, 4-NO_2_Ph, 3-NO_2_Ph, 4-OMePh, Et, 4-NMe_2_Ph, 3,4,5-(OMe)_3_Ph	NH_2_	TBBDA	rt, 30–80 min	88–97	[48]
45	Ph, 3,4,5,-(OMe)_3_Ph, 4-OMePh, 2,3-Me_2_Ph, 4-FPh, 4-ClPh, 2-OHPh, 4-NO_2_Ph, 2,4-Cl_2_Ph, 2-OMePh, 2-ClPh, 2-BrPh, 3-BrPh, 3-FPh, 3-ClPh, 4-(CHO)Ph, Et, 4-CNPh, 4-IPh	Me, Ph	MSI	[Bpy]BF_4_, 80 °C, 25–60 min	82–95	[49]
46	Ph, 4-ClPh, 4-OMePh, 4-MePh, 4-NMe_2_Ph, 4-NO_2_Ph, 2-NO_2_Ph, 2-ClPh, 2,4-Cl_2_Ph, 4-OH-3-OMePh, 3-OMePh, 3-NO_2_Ph, 4-FPh, 2,5-(OMe)_2_Ph, 3,4-(OMe)_2_Ph, 2-MePh, 4-OHPh, 3-ClPh	Me, Ph, NH_2_	succinic acid	120 °C, 3–60 min	65–98	[50]
47	Ph, 4-ClPh, 4-NMe_2_Ph, 3-NO_2_Ph, 2,5-(OMe)_2_	Me, Ph, NH_2_	tannic acid	MW 480 W, 5–13 min	85–90	[51]
48			tannic acid	oil bath, 110–120 °C, 7–20 min	75–90	[51]
49			tannic acid	hot plate, 110–120 °C, 10–21 min	47–76	[51]
50	Ph, 4-ClPh, 4-NMe_2_Ph, 4-MePh, 3-NO_2_Ph, 2,5-(OMe)_2_Ph	Me, Ph, NH_2_	*p*-nitrobenzoic acid	MW 450 W, 8–14 min	82–92	[52]
51			*p*-nitrobenzoic acid	oil bath, 110–120 °C, 12–26 min	80–90	[52]
52			*p*-nitrobenzoic acid	hot plate, 110–120 °C, 13–32 min	60–74	[52]
53	Ph, 4-BrPh, 2-ClPh, 4-ClPh, 2,4-Cl_2_Ph, 4-FPh, 4-OMePh, 4-MePh, 3-NO_2_Ph, 4-NO_2_Ph	Me	CBSA	130 °C, 2–20 min	86–93	[53]
54	Ph, 4-ClPh, 4-OMe, 4-NO_2_Ph, 2-NO_2_Ph, 2-ClPh, 4-MePh	Me, Ph, NH_2_	citric acid	120 °C, 7–43 min	87–94	[54]
55	Ph, 4-ClPh, 4-NMe_2_Ph, 4-MePh, 3-NO_2_Ph, 2,5-(OMe)_2_Ph, 2-thiophene, 1-Nph, 2-Cl-5-FPh	Me, Ph, NH_2_	sulfanilic acid	MW 450 W, 8–14 min	83–94	[55]
56			sulfanilic acid	oil bath, 110–120 °C, 12–24 min	80–95	[55]
57			sulfanilic acid	hot plate, 110–115 °C, 11–28 min	62–72	[55]
58	Ph, 2-NO_2_Ph, 3-NO_2_Ph, 4-NO_2_Ph, 2-ClPh, 4-ClPh, 2,4-Cl_2_Ph, 4-MePh, 4-MeOPh, Et, Pr	Me, Ph, NH_2_	Bi(NO_3_)_3_·5H_2_O	80 °C, 6–150 min	79–97	[56]
59	Ph, 4-ClPh, 4-BrPh, 3-NO_2_Ph, 4-FPh, Et	Me, Ph, NH_2_	1-hexanesulfonic acid sodium salt	MW, 3–20 min	35–95	[57]
60	Ph, 4-NO_2_Ph, 3-NO_2_Ph, 4-OMePh, 4-iPrPh, 2-BrPh, CH_2_-CH_2_-Ph, CH=CH-Ph, C_11_H_23_, 9-phenanthrenyl, 1-pyrenyl	Me, Ph	ZrO(OTf)_2_	80 °C, 1.5–10 min	65–98	[58]
61	Ph, 3-NO_2_Ph, 4-NO_2_Ph, 3-FPh, 4-FPh, 4-OMePh, 2-OMePh	Me, Ph	SO_3_H-carbon	100 °C, 30 min	71–96	[59]
62	Ph, 4-ClPh, 4-BrPh, 4-EPh, 4-MePh, 4-OHPh, 3-OHPh, 4-OMePh, 4-OEtPh, 3-NO_2_Ph, 2-NO_2_Ph, iPr	Me	MCM-41-*N*-propyl- sulfamic acid	130 °C, 90–270 min	35–98	[60]
63	Ph, 2-ClPh, 4-MePh, 3-NO_2_Ph, 2-NO_2_Ph, 4-OMePh, 4-BrPh, 4-ClPh	Me, Ph, NH_2_, NHMe, vinyl	polyphosphate ester	80 °C, 10–20 min	85–93	[61]
64	Ph, 4-MePh, 2-MePh, 4-OMePh, 3-OMePh, 3,4-(OMe)_2_Ph, 4-NMe_2_Ph, 4-NO_2_Ph, 3-NO_2_Ph, 2-NO_2_Ph, 4-ClPh, 2-ClPh, 4-BrPh, 4-FPh, 2,4-Cl_2_Ph	Me	Amberlite IR-120	MW 360 W, 3–6 min	91–96	[62]

Recently the applicability of nanocatalysts in these reactions has been of interest since nanocatalysts, in general, are stable and recyclable and they exhibit higher activity than conventional catalysts. A few notable examples are worth mentioning here. Aluminatesulfonic acid nanoparticles (ASA NPs) proved to be efficient under neat conditions for the synthesis of 1-amidoalkyl-2-naphthols [19]. Zali et al. carried out this synthesis applying nano-sulfated zirconia [20], Borhade et al. used PbS nanoparticles [21], while Safari et al. applied magnetic-nanoparticle-supported sulfuric acid (MNPs-SO_3_H) [22]. As shown in Table 1, entries 2–4, all methods give the desired amidoalkylnaphthols in 77–97% yields. Zolfigol et al. successfully applied 1-methylimidazolium tricyanomethanide {[HMIM]C(CN)_3_} as the first nanostructured molten salt [23]. As depicted in Table 1, entry 5, the catalyst gave remarkable results at room temperature in short reactions (5–30 minutes) in 90–96% yields. Comparing these results with those achieved by the application of tin dioxide nanoparticles (nano SnO_2_, Table 1, entry 6), molten salt catalysis affording higher yields in shorter reactions is definitely more advantageous.

Ionic liquids have also attracted considerable attention due to their „green chemistry” values, including reusability, high thermal stability and non-inflammability. Hajipour et al. reported the one-pot synthesis of 1-amidoalkyl-2-naphthols catalysed by *N*-(4-sulfobutyl)triethylammonium hydrogen sulfate ([TEBSA][HSO_4_]) as Brønsted acidic ionic liquid [24]. In addition, ethylammonium nitrate (EAN) [25], a sulfonic acid-functionalized benzimidazolium-based supported ionic liquid catalyst (SILC) [26], and carboxyl-functionalized benzimidazolium-based ionic liquids (CFBILs) [27] proved to be efficient in the reaction (Table 1, entries 8–10).

Safari et al. combined the benefits of using magnetic nanoparticles and ionic liquids by the application of magnetic Fe_3_O_4_ nanoparticles functionalized with 1-methyl-3-(3-trimethoxysilylpropyl)-1*H*-imidazol-3-ium acetate (MNP-IL-OAc) as catalyst [28]. As shown in Table 1, entries 11 and 12, syntheses carried out by conventional heating at 100 °C required long reaction times affording yields of 82–97%. In contrast, sonication for 10–30 minutes led to improved yields of 90–98%.

There are previous examples for the synthesis of 1-amidoalkyl-2-naphthols carried out in the presence of Lewis and Brønsted acid catalysts. As depicted in Table 1, entries 13–38, the applicability of *p*-toluenesulfonic acid (*p*-TSA) [27], montmorillonite K10 [30], Indion-130 [31], iodine (I_2_) [32], potassium dodecatungstocobaltate (K_5_CoW_12_O_40_·3H_2_O) [33], silica-supported perchloric acid (HClO_4_-SiO_2_) [34–36] and sulfuric acid [35], chlorosulfonic acid [38], sodium hydrogen sulfate (NaHSO_4_·H_2_O) [39], ferric(III) hydrogen sulfate [Fe(HSO_4_)_3_; 40], strontium(II) triflate, Sr(OTf)_2_; [41], copper-exchanged heteropoly acids, Cu_1·5_PMo_12_O_40_ (CuPMo) and Cu_1·5_PW_12_O_40_ (CuPW); [42] or wet cyanuric acid (wet-TCT) [43] was also tested. These methods suffer from a number of drawbacks, such as strong acidic media, high temperature, and prolonged reactions. Furthermore, the yields are often not satisfactory.

To eliminate the disadvantages of previous strategies, Samant et al. reported an ultrasound-promoted condensation catalysed by sulfamic acid [44]. As shown in Table 1, entries 39 and 40, both dichloroethane (DCE) and solvent-free conditions were tested. The catalyst worked at low temperature (28–30 °C) and the products were formed in short reaction times in up to 94% yields. Shinde et al. also published iodine catalysis carried out at room temperature in DCE [45]. Whereas long reaction times were needed in the latter process, good yields could be achieved under mild conditions.

In additional publications listed in Table 1, entries 42–64, phosphorus pentoxide (P_2_O_5_) [46], silica-supported phosphorus pentoxide (P_2_O_5_-SiO_2_) [47], *N*,*N*,*N*’,*N*’-tetrabromobenzene-1,3-disulfonamide (TBBDA) [48], 1-methyl-3-(2-(sulfoxy)ethyl)-1*H*-imidazol-3-ium chloride (MSI) [49], succinic acid [50], tannic acid [49], *p*-nitrobenzoic acid [52], a carbon-based solid acid (CBSA) [53], citric acid [54], sulfanilic acid [55], bismuth(III) nitrate pentahydrate (Bi(NO_3_)_3_·5H_2_O) [56], 1-hexanesulfonic acid sodium salt [57], zirconyl triflate (ZrO(OTf)_2_) [58], sulfonated carbon (SO_3_H-carbon) [59], MCM-41-*N*-propylsulfamic acid [60], polyphosphate ester [61] and amberlite IR-120 [62] were used as catalysts. These latest strategies provide efficient syntheses under mild conditions without using harsh chemicals. Furthermore, the application of microwave irradiation or sonication is also preferred to conventional heating methods to accelerate the reactions.

#### Synthesis of aminoalkylphenols

The mechanism of the formation of phenolic Mannich bases is similar to that discussed above for the synthesis of amidoalkylnaphthols. First, the phenol component reacts with the aldehyde to form the *o*-QM intermediate, which reacts in a nucleophilic addition step with the amine component, resulting in aminoalkylphenol derivatives. A few examples are summarized in Table 2. An important difference although must be noted. In the case of aminonalkylnaphthols, the *o*-QM intermediate partially remains aromatic while the formation of phenolic *o*-QMs leads to the loss of the aromaticity of the only aromatic ring present. This results in differences in both the formation and stability of *o*-QM.

**Table 2:** Formation and substrate scope of phenolic Mannich bases.

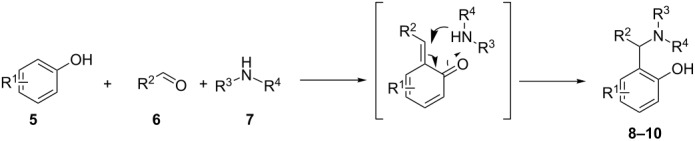					

entry	R^1^	R^2^	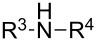	product	reference

1	4-Me, 4-COOMe	Ph, 4-MeOPh, 4-NO_2_Ph, 3-CF_3_Ph, 2-All-O-Ph	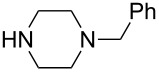	**8a**–**i**	[63]
2	H, 2-Me, 3,5-(OH)_2_-2,4-(CHO)_2_, 3,5-(OH)_2_-2,4-Ac_2_, 3,5-(OH)_2_-2,4-dipropanoyl, 3,5-(OH)_2_-2,4-diisobutanoyl, 3,5-(OH)_2_-2,4-dibutanoyl	H	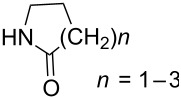	**9a**–**t**	[64–65]
3	3-Cl, 2-NO_2_, 4-OMe, 4-NO_2_, 4-Cl, 2,4-Cl_2_	4-BrPh, 4-NO_2_Ph, 4-ClPh, 4-OMePh, 2,3-Me_2_Ph, 4-*t*-BuPh	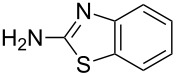	**10a**–**m**	[66]

Grimaud et al. synthesized Mannich bases starting from *N*-benzylpiperazine, various aldehydes and substituted phenols [63]. The intermediate Mannich adducts **8a**–**i** were then reacted with 5,5-dimethylcyclohexane-1,3-dione or 1-methyl-1*H*-indole in the presence of lithium perchlorate as catalyst to afford the new 3,3-dimethyl-2,3,4,9-tetrahydro-1*H*-xanthen-1-ones and 3-substituted indoles. The process was then extended to isocyanides and new aminobenzofurans formed via [4 + 1] cycloaddition were isolated.

Bharate et al. reported *ortho*-amidoalkylation of phenols in which a tandem Knoevenagel condensation occurs through *o*-QM followed by the formation of an unstable oxazine intermediate [64]. Later, the same research group published a similar reaction extended by various lactams carried out in trifluoroacetic acid in water [65]. As reported in both papers, Mannich bases formed **9a**–**t** were isolated in good yields. Plausible reaction pathways were described and the energetic values of the transition states were calculated.

In one of the latest publications with respect to this topic, Priya et al. disclosed the synthesis of a wide range of novel 2-[(benzo[*d*]thiazol-2-ylamino(phenyl)methyl]phenols **10a**–**m** [66]. In their study, 2-amino-1,3-benzothiazoles, various aldehydes and substituted phenols were reacted in the presence of ZnCl_2_ as catalyst.

### Reactions of *o*-QMs formed by Mannich bases

As the formation of Mannich bases can be explained by the generation of an *o*-QM intermediate followed by the nucleophilic addition of the amine component, the reverse reaction with the corresponding nucleophile is also feasible. Mechanistically, the Mannich adduct generates an *o*-QM via the loss of an amine, then this reactive intermediate reacts with the nucleophile (dienophile) species in different reactions to form a wide range of heterocyclic compounds.

#### Reactions with C=C dienophiles

Reactions of *o*-QMs with different C=C dienophiles are listed in Table 3. Osyanin et al. reported the efficient reaction of quaternary ammonium salt Mannich bases with malononitrile catalysed by 1,8-diazabicyclo[5.4.0]undec-7-ene (DBU) [67]. It is known that the use of quaternary ammonium salts offers the easier removal of the amino residue and, therefore, trapping the transient electrophilic species at lower temperature. Carrying out the reactions in protic solvents such as H_2_O or EtOH at 100 °C, the desired products were formed in short reactions (1–20 min) and chromene-2-carbonitriles **12**–**15** were isolated in 61–88% yields.

**Table 3:** Reactions of *o*-QMs with different dienophile species.

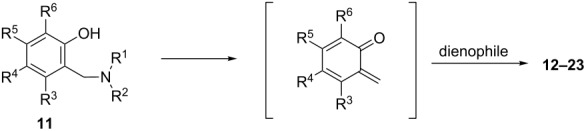							

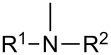	R^3^	R^4^	R^5^	R^6^	dienophile	product	ref.

NMe_3_^+^	H	Me, Ad, *t-*Bu, Ac, Bn, Cl	H	H	CH_2_(CN)_2_	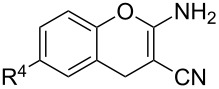 **12**	[67]
NMe_3_^+^	H	Me	H	Ad	CH_2_(CN)_2_	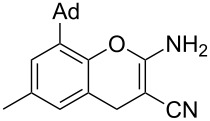 **13**	[67]
NMe_3_^+^	H	H	Ac	H	CH_2_(CN)_2_	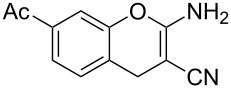 **14**	[67]
NMe_3_^+^	H	Me	Me	H	CH_2_(CN)_2_	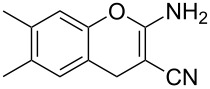 **15**	[67]
NMe_2_, NEt_2_, 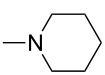	H	H, Ph	H, Ph	H, Ph	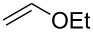	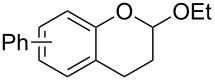 **16**	[68]
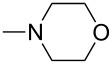	OH	H	H	COMe	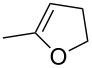	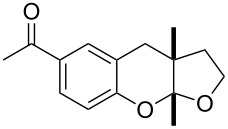 **17**	[69]
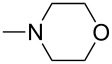	OH	H	H	COMe	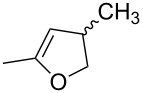	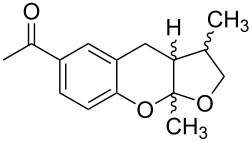 **18**	[69]
NBn_2_	H	MeO	iPr	MeO	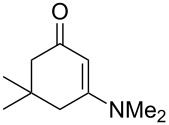	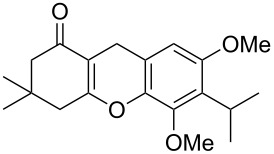 **19**	[70]
NMe_3_^+^	H	H, Br, COMe, 1-Ad, NO_2_, *t-*Bu, Me	H	H, Br, 1-Ad, NO_2_	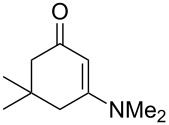	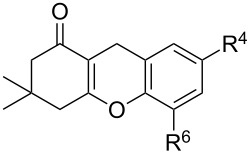 **20**	[71]
NMe_3_^+^	H	H, Me, COMe, CO_2_Me, NO_2_	H, Me, CO_2_Me	H, OMe	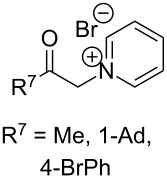	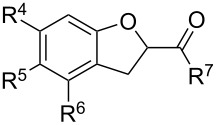 **21**	[72]
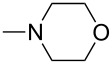	H	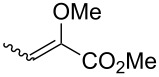	CO_2_Me	OMe	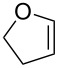	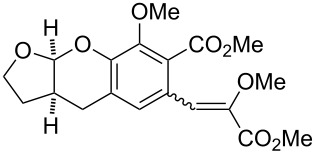 **22**	[73]
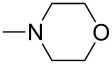	H	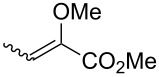	CO_2_Me	OMe	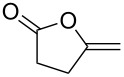	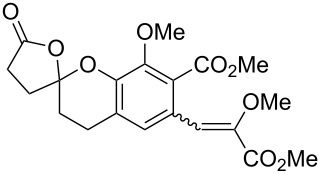 **23**	[73]

Saito et al. generated *o*-QMs starting from Mannich bases by low-energy UV irradiation in aqueous acetonitrile [68]. In the presence of a large excess of ethyl vinyl ether as *o*-QM trapping agent, they isolated several 2-ethoxychromane derivatives **16**. However, yields as low as 36% were found in some cases.

*o*-QMs derived from Mannich adducts also appear to be key intermediates in the syntheses of biologically active natural compounds. Wilson et al. successfully achieved the total synthesis of xyloketals **17** and **18**, including cycloaddition of substituted dihydrofurans and 1-(2,4-dihydroxy-3-(morpholinomethyl)phenyl)ethanone via *o*-QM intermediates [69]. Osyanin et al. reported the synthesis of *Uvaria scheffleri* alkaloids espintanol and (±)-schefflone starting from 6-((dibenzylamino)methyl)-3-isopropyl-2,4-dimethoxyphenol. The *o*-QM formed be trapped by 3-(dimethylamino)-5,5-dimethylcyclohex-2-en-1-one resulting in tetrahydro-1*H*-xanten-1-one **19** [70]. Starting from phenolic Mannich bases and 3-dimethylamino-2-cyclohexen-1-ones, the synthesis of 2,3,4,9-tetrahydro-1*H*-xantene-1-ones (**20**) has been published by the same research group. The synthetic protocol was then extended to isolate benzo[*a*]xanthen-11-ones or chromeno[3,2-*g*]β-carboline-8,13-dione starting from 2-naphthol and 1*H*-β-carboline-1-one Mannich bases [71]. Although a high temperature was needed (reflux at 153 °C for 4 hours), the desired products were isolated in good (53–91%) yields. The authors reported better results with the use of polyheterocyclic initial compounds. This can be explained by a dearomatisation step taking place in the transformation of phenolic Mannich bases, leading to the disappearance of the only aromatic ring. In a recent publication by same research group [72], they elaborated a simple route to 1,2-dihydronaphtho[2,1-*b*]furan and 2,3-dihydrobenzofurans via base-induced desamination. They also reported the development of a simple, general route to 2,3-dihydrobenzofurans **21** starting from phenolic Mannich bases. The syntheses were also extended to 2-naphthol Mannich bases as initial compounds affording C-2-substituted 1,2-dihydronaphtho[2,1-*b*]furans.

Bray et al. reacted *ortho*-hydroxybenzylamines with 2,3-dihydrofuran and γ-methylene-γ-butyrolactone in DMF at 130 °C [73]. This method could successfully be applied in the synthesis of the spiroketal core of rubromycins **22** and **23**.

**Scheme 2:**
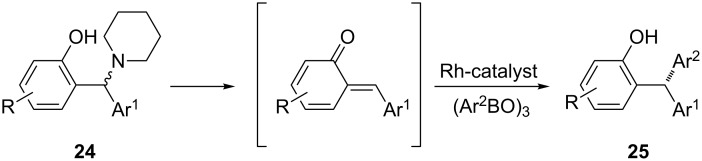
Asymmetric syntheses of triarylmethanes starting from diarylmethylamines.

One of the latest publications around the topic is published by Hayashi et al. in 2015 [74]. Starting from diarylmethylamines **24** and arylboroxines, they successfully developed a rhodium-catalyzed asymmetric arylation process leading to triarylmethanes **25**. With the application of mild reaction conditions (40 °C, 15 h), a high enantioselectivity (≥90% ee) was reached with good to excellent yields. (Scheme 2).

**Scheme 3:**
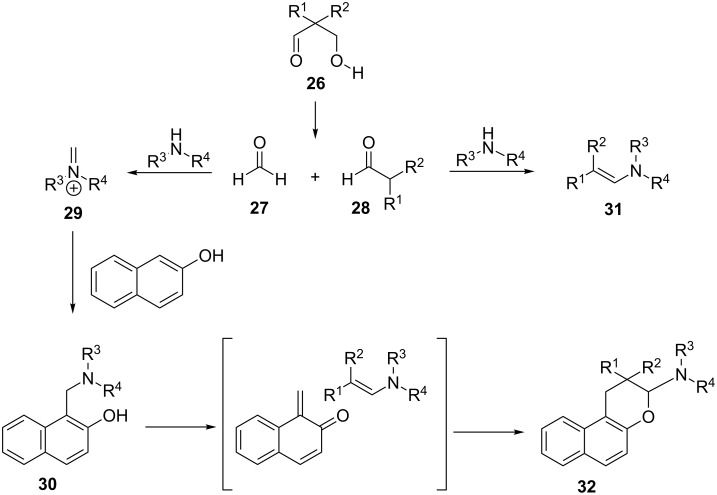
Proposed mechanism for the formation of 2,2-dialkyl-3-dialkylamino-2,3-dihydro-1*H*-naphtho[2,1-*b*]pyrans **32**.

Starting from 2-naphthol, 2,2-disubstituted 3-hydroxypropanals and cyclic secondary amines, Jha et al. achieved the synthesis of 2,2-dialkyl-3-dialkylamino-2,3-dihydro-1*H*-naphtho[2,1-*b*]pyrans under solvent-free conditions using *p*TSA as catalyst [75]. It is important to note, that during the reaction, 2,2-disubstituted 3-hydroxypropanals **26** decompose to formaldehyde and 2,2-disubstituted acetaldehydes **28**. Formaldehyde, as a non-enolizable compound is more likely to give Mannich base product **30**. In contrast, enolizable 2,2-disubstituted aldehydes easily form enamines **31** that undergo cycloaddition with electron-deficient *o*-QMs giving 2,2-dialkyl-3-dialkylamino-2,3-dihydro-1*H*-naphtho[2,1-*b*]pyrans **32**. A plausible mechanism is depicted in Scheme 3.

**Scheme 4:**
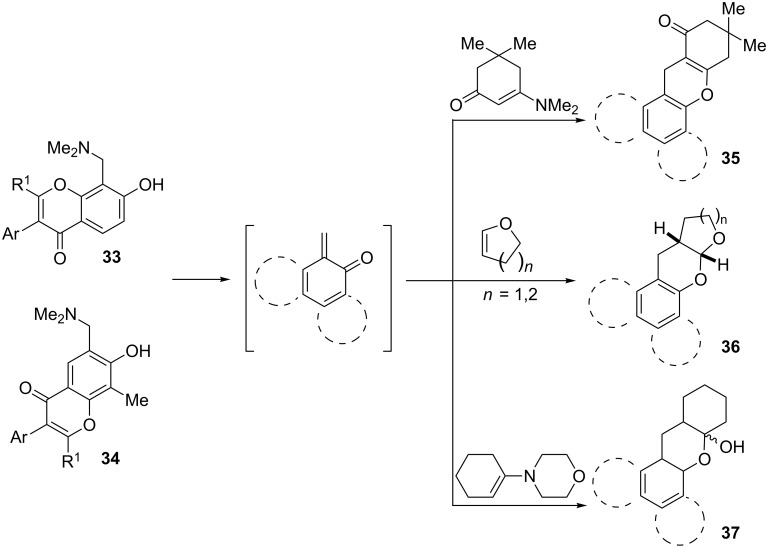
Cycloadditions of isoflavonoid-derived *o*-QMs and various dienophiles.

Watt et al. achieved the regioselective condensation of bis(*N*,*N*-dimethylamino)methane with various hydroxyisoflavonoids to synthesize C-6- and C-8-substituted isoflavonoids **33** and **34** in a Mannich-type reaction [76]. These *o*-QM precursors by a thermal elimination of dimethylamine were then reacted with different cyclic dienophiles to give various inverse electron-demand Diels–Alder adducts **35**–**37**. In case of **36**, the *cis*-fused ring system found to be similar to bioactive xyloketals isolated from fungi (Scheme 4)

*o*-QMs are also known to undergo oligomerization in the absence of dienophiles and nucleophiles via an oxo-Diels–Alder protocol (Table 4). During the syntheses of 1,4,9,10-anthradiquinones with potential antitumor activity, Kucklaender et al. isolated new spiro derivatives **38** [77]. These latter spirocyclic dimers formed in a Diels–Alder dimerization process by heating the corresponding Mannich bases under reflux in dichloromethane for 2 hours.

**Table 4:** Dimerization of *o*-QMs.

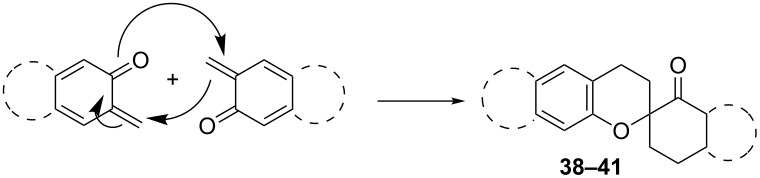			

entry	monomer	dimer	reference

1	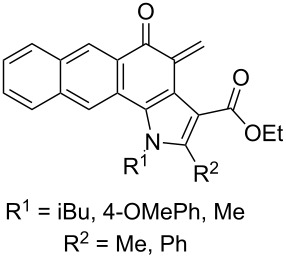	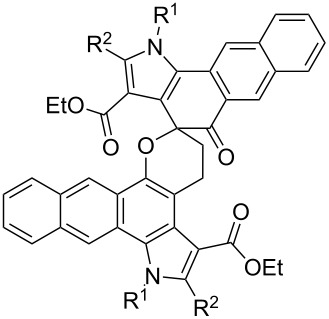 **38**	[77]
2	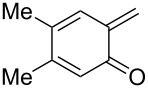	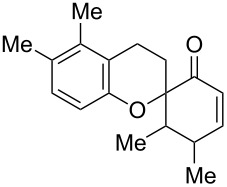 **39**	[78]
3	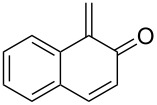	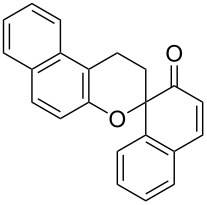 **40**	[78]
4	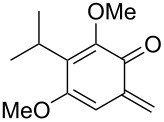	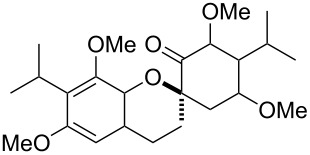 **41**	[71]

In the synthesis of 3,4-dihydro-2-aryl-2*H*-benzo[*f*]chromenes and 2-aryl-6,7-dimethylchromans starting from substituted styrenes and 1-dimethylaminomethyl-2-naphthol or 2-dimethylaminomethyl-4,5-dimethylphenol, Bilgiç et al. detected the formation of both dimers **39** and **40** of *o*-QMs formed by the thermal desamination of the initial compounds [78]. However, some of the publications report this phenomenon as an advantageous reaction rather than the formation of unexpected side products. As mentioned above [71], Osyanin et al. reported the synthesis of *Uvaria scheffleri* alkaloid (±)-schefflone. In this publication, the dimerization of the *o*-QM resulting in intermediate **41** is a key step in the synthesis of the final natural trimer compound.

#### Reactions with C=N dienophiles

**Scheme 5:**
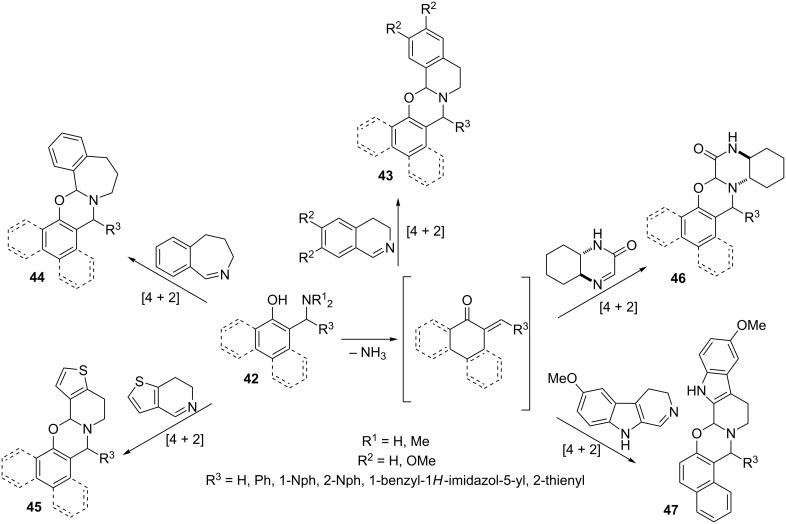
[4 + 2] Cycloaddition reactions between aminonaphthols and cyclic amines.

The preparation of novel *o*-QM-condensed poliheterocycles is a relatively new area of Mannich base chemistry. Our research group has also been interested in cycloaddition reactions of *o*-QMs generated from Mannich adducts **42**, when a serendipitous reaction occurred. Namely, the formation of new naphthoxazino-isoquinoline derivatives **43** under neat conditions staring from 1-aminoalkyl-2-naphthols and 6,7-dimethoxy-3,4-dihydroisoquinoline was observed [79]. At the same time, Osyanin et al. reported the same reaction extended by various substituted aminonaphthols [80]. Achieving the syntheses in ethanol at 78 °C, [4 + 2] cycloaddition took place between the *o*-QM generated from the corresponding aminonaphthol as diene component and cyclic imines playing the role of heterodienophiles (Scheme 5).

Fülöp and co-workers then extended their studies by applying both 2-aminoalkyl-1-naphthols and 1-aminoalkyl-2-naphthols [81]. These bifunctional compounds were reacted with various cyclic imines such as 4,5-dihydrobenzo[*c*]azepine or 6,7-dihydrothieno[3,2-*c*]pyridine to have new naphthoxazinobenzazepine **44** and -thienopyridine **45** derivatives [82]. Transformations at 80 °C in 1,4-dioxane as solvent were performed in a microwave reactor to utilize the advantages of this method. As expected, reaction times shortened, while the products were isolated in higher yields in comparison with those found by conventional heating.

The application of (4a*S*,8a*S*)-hexahydroquinoxalin-2-one served as the first example with respect to the use of an enantiomeric cyclic imine in this type of reaction [83]. The formation of the possible naphthoxazino-quinoxalinone diastereomers **46** was investigated and studied by theoretical calculations (Scheme 5). In this and all previous cases, the conformational behaviour of the polyheterocycles formed was also described.

The [4 + 2] cycloadditions between cyclic imines and *o*-QMs derived from Mannich bases could also be successfully applied in the syntheses of natural alkaloid-like compounds **47**. Osyanin et al. reported the preparation of rutaecarpine and evodiamine, the 14-oxa analogues of *Evodia rutaecarpa* alkaloids, starting from 6-methoxy-4,9-dihydro-β-carboline and various substituted 1-aminoalkyl-2-naphthols [84] (Scheme 5).

#### Reactions with electron rich aromatic compounds

**Scheme 6:**
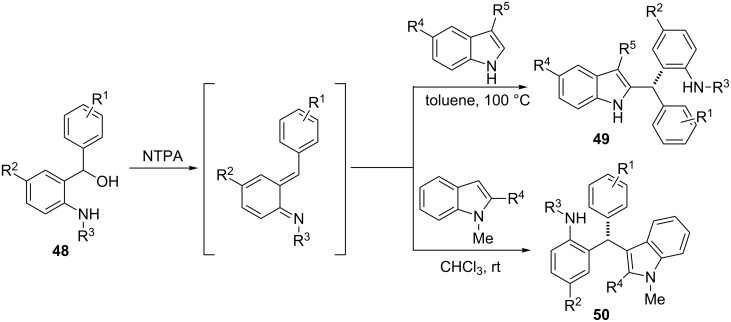
Brønsted acid-catalysed reaction between aza-*o*-QMs and 2- or 3-substituted indoles.

The formation of aza-*o*-QMs is also possible, if the initial phenolic Mannich base bears an aromatic moiety on its benzylic carbon atom. Rueping et al. recently performed reactions between aza-*o*-QMs in situ generated from α-substituted *ortho*-amino benzyl alcohols **48** and substituted indoles catalysed by *N*-triflylphosphoramides (NTPAs) [85]. (Scheme 6) The process provided new C-2 and C-3-functionalized indole polyheterocycles **49** and **50** in good yields with 90–99% ee.

**Scheme 7:**
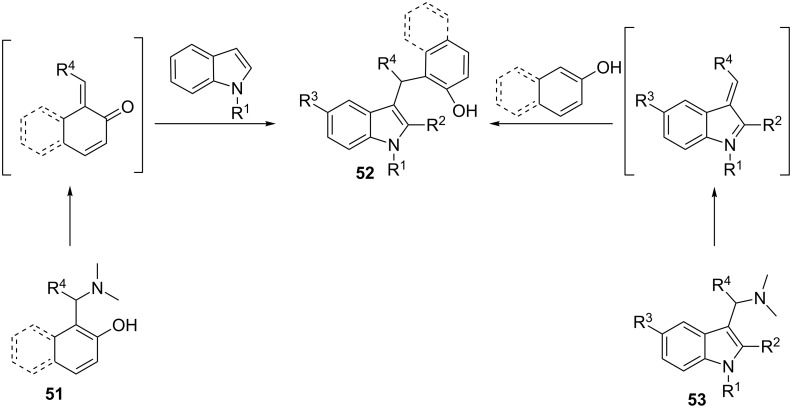
Formation of 3-(α,α-diarylmethyl)indoles **52** in different synthetic pathways.

One of the latest publications around this topic has been reported by Deb et al. [86–87]. Various 2-(aminoalkyl)phenols or 1-(aminoalkyl)naphthols **51** were reacted with indoles under Brønsted acid catalysis resulting in 3-(α,α-diarylmethyl)indoles **52**. Then, through C-2 cyclization of the indole ring using I_2_ as catalyst and *tert*-butyl hydroperoxide as oxidant, chromeno[2,3-*b*]indoles were isolated in 71–98% yields. In a different reaction pathway, starting from 3-(aminoalkyl)indoles **53** and phenols or naphthols, 3-(α,α-diarylmethyl)indoles **52** were also formed in around 90% yields under microwave irradiation (Scheme 7).

#### Miscellaneous reactions

It is also known that *o*-QMs could cross-link two biologically important molecules such as peptides, proteins or nucleic bases. (see section Biological properties) Achieving the reaction under physiological conditions, it is possible to extend the syntheses to biomolecular applications.

**Scheme 8:**
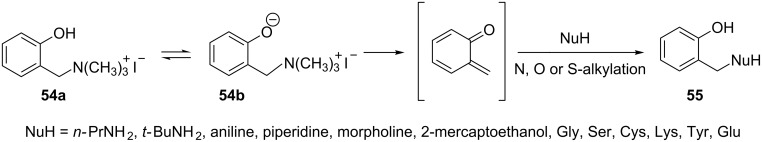
Alkylation of *o*-QMs with N-, O- or S-nucleophiles.

Starting from (2-hydroxybenzyl)trimethylammonium iodide (**54**), Freccero et al. successfully trapped *o*-QMs formed by several N*-*, O*-* and S*-*nucleophiles [88]. They examined both thermal and photochemical generations of such intermediates. By selecting the appropriate reaction conditions (various pH and temperatures), they were able to alkylate free amino acids, e.g., glycine (Gly), L-serine (Ser), L-cysteine (Cys), L-lysine (Lys), L-tyrosine (Tyr) and glutathione (Glu) in aqueous solution to isolate **55** (Scheme 8).

**Scheme 9:**
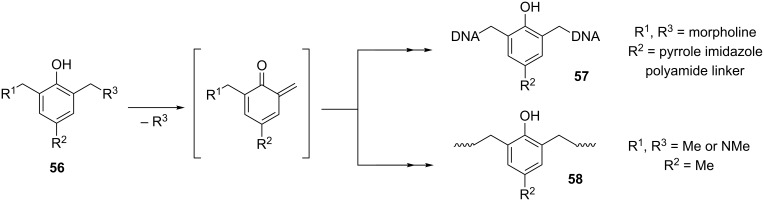
Formation of DNA linkers and *o*-QM mediated polymers.

Rokita et al. focused on generating *o*-QMs and used them as cross-linking and DNA alkylating agents. Starting from Mannich base **56** and transforming it by a number of synthetic steps, they were managed to elaborate a process that provides easy access to *o*-QM precursors containing a broad array of linkers **57**, which were used to connect with site-directing ligands [89] (Scheme 9).

As reactive intermediates, *o*-QMs can also play the role of monomers in polymerization reactions. Ishida et al. reported the ring-opening polymerization of monofunctional alkyl-substituted aromatic amine-based benzoxazines [90]. It was shown that the methylene bridges can be formed by *o*-QMs that are resulted by the cleavage of phenolic Mannich bridge structure **56** (Scheme 9).

### Biological properties

As discussed earlier, *o*-QMs are known as short-lived, highly reactive intermediates. Therefore, their biological activity is mostly examined from the point of view of their application as DNA alkylating agents. One of the first examples was reported by Kearney et al. in 1996 about preformulation studies of the antitumor agent topotecan [91]. The antitumor activity of the compound could be explained by its degradation to highly active zwitterionic species via an *o*-QM intermediate. Dimmock et al. subsequently examined the cytotoxic activity of phenolic azobenzene Mannich bases [92]. Correlations were found between structures and activities against murine P388DI and L1210 cells, human T-lymphocyte cell lines and, in some cases, mutagenous properties were also shown.

Freccero et al. examined the photogeneration by laser flash photolysis and reactivity of naphthoquinone methides as well as their activity as purine selective DNA alkylating agents [93]. Farrell et al. studied the mechanism of the cytotoxic action of naphthoquinone–platinum(II) complexes [94]. Both DNA binding and topoisomerase I inhibition studies proved that the coordination and stabilization of the quinone methide structure can effect marked changes in DNA reactivity. In a recent publication, 3-(aminomethyl)naphthoquinones were investigated from the point of view of cytotoxicity, structure–activity relationships and electrochemical behaviour [95]. Derivatives that contain an aromatic amine and salicylaldehyde or 2-pyridinecarboxaldehyde moieties were found to be the most active against the HL-60 (promyelocytic leukaemia) cell line. Zhou et al. obtained phenolic Mannich bases bearing functional groups that are suitable for cross-linking DNA; therefore, their antitumor effects could also be confirmed [96].

The formation of *o*-QMs and their biological properties were also illustrated by kinetic studies. Rokita et al. using laser flash photolysis showed that formation and reactivity of these intermediates strongly depended on the presence of electron-donating or electron-withdrawing functional groups of the *o*-QM precursors [97].

## Conclusion

The high number of publications that has recently appeared on the *o*-QM-mediated Mannich-type transformations is a clear indication that the application of this highly-reactive intermediate has made the modified Mannich reaction to be a hot topic again in organic chemistry. This review presents a wide range of applications including cycloadditions and the synthesis of bifunctional amino- or amidonaphthols that can later be transferred as building blocks into several natural or biologically active compounds. Thanks to the immense number of possibilities for Mannich reaction through the use of various amines, aldehydes and electron-rich aromatic compounds, the continued evolution of the literature on these reactions appears to be guaranteed. By the application of various cyclic imines and subsequently extended by the use of nonracemic derivatives, a wide range of enantiomeric polyheterocyclic compounds could be isolated and might be tested as potential anticancer drug candidates.
